# Cholangiocarcinoma With Rare Esophageal Metastasis

**DOI:** 10.14309/crj.0000000000000717

**Published:** 2022-01-10

**Authors:** Mana Matsuoka, Katsumasa Kobayashi, Yukito Okura, Takahito Nozaka, Ayako Sato, Masato Yauchi, Taichi Matsumoto, Yohei Furumoto, Takao Horiuchi, Toru Asano

**Affiliations:** 1 Department of Gastroenterology, Tokyo Metropolitan Bokutoh Hospital, Sumida-ku, Tokyo, Japan

## CASE REPORT

**Figure 1.**
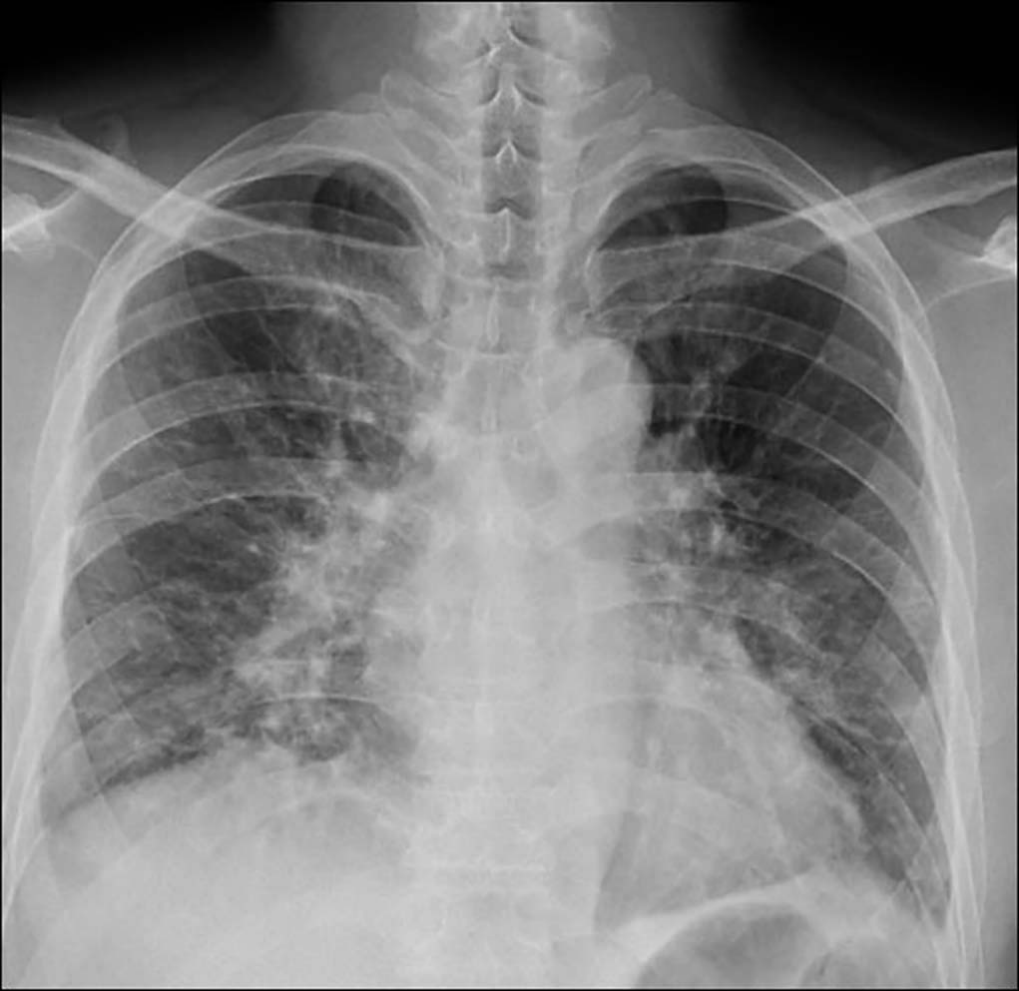
Chest radiograph showing diffuse ground-glass opacities in both lung fields.

**Figure 2.**
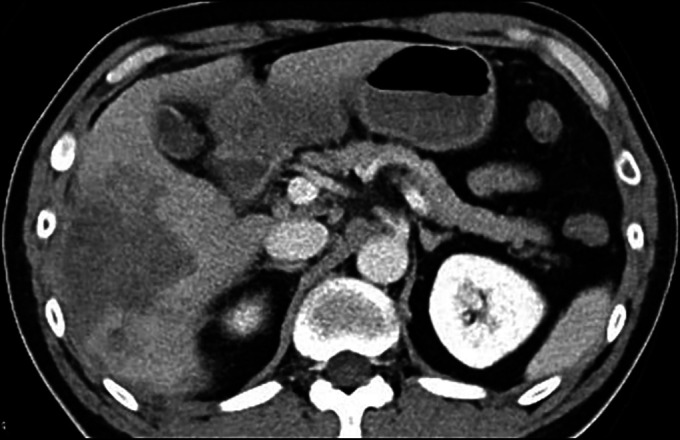
Contrast-enhanced computed tomography showing an irregular, well-defined, poorly enhanced tumor, measuring approximately 70 mm in diameter, in the right lobe of the liver.

A 49-year-old man with no relevant medical history presented with a 2-week history of dyspnea. His respiratory rate was 20 breaths/minute, and his saturation of percutaneous oxygen decreased to 88% in room air. A chest radiograph showed diffuse ground-glass opacities in both lung fields (Figure 1). His serum carbohydrate antigen 19–9 level was abnormally high at 36,208.6 U/mL. Contrast-enhanced computed tomography showed an irregular, well-defined, poorly contrast-enhanced tumor, approximately 70 mm in diameter, in the right lobe of the liver, suggestive of an intrahepatic cholangiocarcinoma (Figure 2). Furthermore, lymphadenopathy was observed at the hepatic hilum.

**Figure 3.**
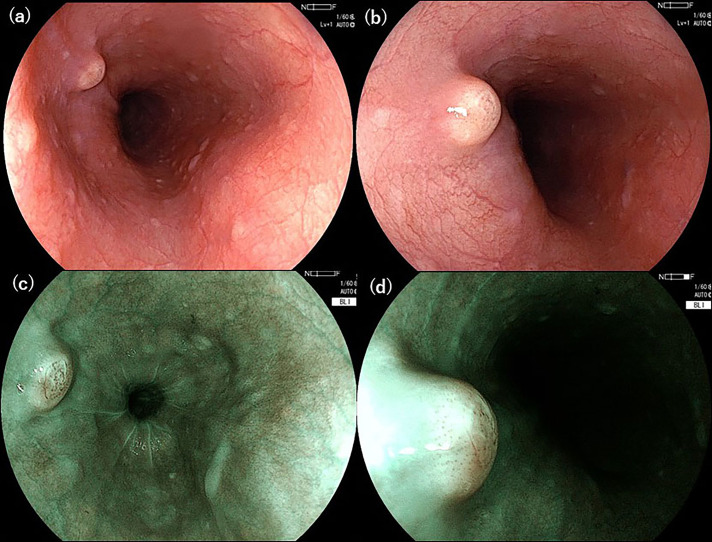
(A and B) Esophagogastroduodenoscopy shows a single, white-toned, hard tumor of 5 mm diameter, which is shaped like a submucosal tumor (SMT), 30 cm from the incisor. (C and D) Atypical blood vessels of different caliber are seen on top of the tumor in a magnified observation using blue laser imaging.

**Figure 4.**
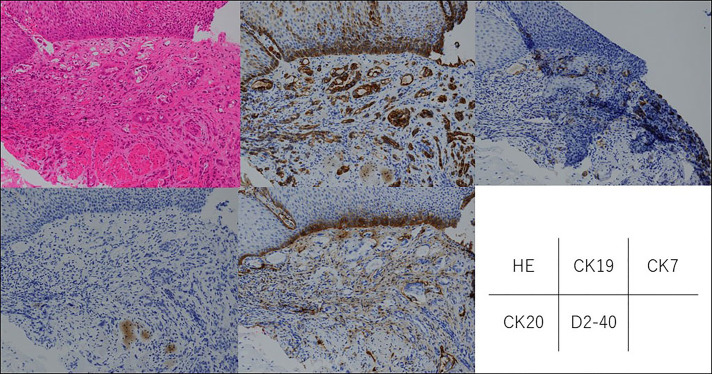
Histopathological section shows an invasive, poorly differentiated adenocarcinoma just below the esophageal squamous epithelium (positive staining for cytokeratin [CK] 7 and CK 19, and negative for CK 20, which is consistent with cholangiocarcinoma). In addition, floating cancer follicles in the lymphatic vessels, which stained positive for D2-40, were suggestive of lymphatic metastasis.

Esophagogastroduodenoscopy, which was performed to determine the presence of other primary lesions, showed a single, white-toned, hard tumor measuring 5 mm in diameter, shaped like a submucosal tumor, 30 cm from the incisor (Figure 3). By magnified observation using blue laser imaging, atypical blood vessels were observed to be distributed on the top of the tumor (Figure 3). Endoscopic biopsies revealed an invasive, poorly differentiated adenocarcinoma just below the esophageal squamous epithelium (positive staining for cytokeratin 7 and CK19, and negative staining for CK20, which is consistent with a cholangiocarcinoma). In addition, floating cancer follicles were noted in lymphatic vessels, which stained positive for D2-40, suggesting the presence of lymphatic metastasis (Figure 4). Bronchoscopy showed edema of the bronchial lumen in the absence of metastasis. Transbronchial lung biopsy demonstrated the infiltration of inflammatory cells, whereas no tumor cells were detected. Percutaneous liver tumor biopsy could not be performed because the tumor was in contact with the liver's surface, although no other primary tumor was found. Therefore, the patient was diagnosed with intrahepatic cholangiocarcinoma and lymphangitis carcinomatosa. Although he underwent chemotherapy with gemcitabine, cisplatin, and S-1, the cancer progressed rapidly. He died on the 23rd day of hospitalization.

Malignant tumors rarely metastasize to the esophagus through hematogenous or lymphatic routes. Only 1 case of esophageal metastasis from a cholangiocarcinoma has been reported.^1^ In that case, the tumor was pedunculated, with multiple lesions and ulcerations. Metastatic esophageal tumors usually have multiple longitudinal runs of short nodules or SMT-like shapes.^2^ Unusually, the tumor in the patient from the previous report had a single, small SMT-like shape because of the mode of cancer metastasis.^1^ The diagnostic rate of biopsy for submucosal tumor is typically low; however, atypical findings on magnified observation can assist accurate diagnosis.^3^

## DISCLOSURES

Author Contributions: M. Matsuoka wrote the manuscript and is the article guarantor. K. Kobayashi wrote and critically reviewed the manuscript. Y. Okura, T Nozaka, A. Sato, M. Yauchi, T. Matsumoto, Y. Furumoto, T. Horiuchi, and T. Asano critically reviewed the manuscript. All authors approved the final version of the manuscript.

Financial disclosures: None to report.

Informed consent was obtained for this case report.
